# Derivation of the non-inferiority margin for the evaluation of direct oral anticoagulants in the treatment of venous thromboembolism

**DOI:** 10.1186/1477-9560-11-13

**Published:** 2013-07-06

**Authors:** Martin H Prins, Anthonie WA Lensing

**Affiliations:** 1 Maastricht University Medical Center, Maastricht, The Netherlands; 2 Bayer Pharma AG, Wuppertal, Germany

**Keywords:** Non-inferiority margin, Oral anticoagulants, Venous thromboembolism

## Abstract

**Background:**

Direct oral anticoagulants that target a single coagulation factor have been developed as an alternative to standard therapies with heparin and/or vitamin K antagonists. The purpose of this study was to derive non-inferiority margins suitable for randomised clinical studies designed to evaluate these agents for the treatment of venous thromboembolism (VTE).

**Methods:**

We performed a systematic review to derive non-inferiority margins suitable for use in studies evaluating direct oral anticoagulants for the treatment of VTE. A PubMed search identified publications that evaluated current standard treatment versus placebo, ‘no treatment’ or ‘less intensive treatment’ in patients with symptomatic deep vein thrombosis (DVT) and/or pulmonary embolism (PE). Publications were eligible if they had a randomised study design, included patients with symptomatic DVT and/or PE, used objective diagnostic methods to document the index event and reported objectively confirmed symptomatic recurrent VTE.

**Results:**

Fourteen publications were included in the analysis. Recurrent VTE occurred in 25 (1.5%) out of 1715 patients who received current standard of care and in 157 (9.2%) out of 1711 patients who received placebo, ‘no treatment’ or ‘less intensive treatment’, for an odds ratio of 0.18 (95% confidence interval, 0.14−0.25; test for heterogeneity, p=0.87). In order to preserve 50% or 75% of the established treatment effect using a linear scale, the corresponding thresholds for non-inferiority equalled 2.50 and 1.75, respectively.

**Conclusions:**

This systematic review and statistical approach determined non-inferiority margins suitable for use in studies of direct oral anticoagulants for the treatment of DVT and/or PE.

## Background

Adjusted-dose unfractionated heparin or bodyweight-adjusted low molecular weight heparin overlapping with and followed by laboratory-titrated vitamin K antagonists (VKAs) is widely accepted as the standard treatment for patients presenting with acute deep vein thrombosis (DVT) and/or pulmonary embolism (PE) [1]. Although highly efficacious and relatively safe, this treatment regimen is associated with a considerable number of disadvantages, including the need for parenteral administration of heparin and frequent laboratory monitoring of the pharmacodynamic effects of VKAs with subsequent dose adjustments.

Recently, small molecules have been developed that directly inhibit a single component of the coagulation cascade (such as Factor Xa or thrombin), can be taken orally and have a predictable pharmacokinetic/pharmacodynamic profile [2-4]. Although this eliminates the need for parenteral administration and routine coagulation monitoring, it is essential that these new compounds are demonstrated to be clinically as efficacious as the current standard treatment. Demonstration of similar efficacy requires randomised trials involving patients with symptomatic DVT and/or PE that compare the incidences of recurrent venous thromboembolism (VTE) between the new compounds and the current standard treatment. To exclude a clinically relevant excess of recurrent VTE, the direct oral anticoagulants need to satisfy a non-inferiority margin that is based on the documented efficacy of current standard treatments [5,6].

In this paper, we systematically reviewed all studies that have evaluated the efficacy of the current standard treatment for DVT and/or PE and calculated accompanying non-inferiority margins from the meta-analysis.

## Methods

### Literature search

A PubMed search was undertaken for articles reporting the results of controlled randomised trials between the dates of 1959 and 2013 to identify studies that compared the currently recommended anticoagulant treatment approach with placebo, ‘no treatment’ or a ‘less intensive’ heparin/VKA regimen. The search relied on titles, abstracts and multiple database descriptors and was not restricted by language. The following key words were used to identify potential relevant publications and eligible studies for inclusion from a search of MEDLINE: heparin or low molecular weight heparin, venous thrombo*, vitamin K antagonist, warfarin, acenocoumarol, phenprocoumon and fluindione, and the publications were limited to randomised controlled trials. This broad search approach was followed by an extensive manual search. Reports were eligible if they included a randomised study design, included patients with symptomatic DVT and/or PE, used objective diagnostic methods to document the index event and reported objectively confirmed symptomatic recurrent VTE. The early study of Barritt and Jordan in 1960 is, in fact, the only ‘no-treatment controlled’ study and treated patients for 14 days only [7]. Other early studies dated between 1979 and 1993 addressed mainly the initial 3 months of treatment and used ‘less intensive’ heparin therapy and/or VKA treatment [8-13]. Studies published since 1993 addressed mainly the need for continuation of VKA therapy and compared this therapy to ‘no treatment’ or placebo treatment in the initial 4 weeks to 6 months after the index event [14-20]. All studies involving ‘less intensive’ regimens that were demonstrated to be superior versus placebo or ‘no treatment’ were excluded.

### Data extraction

The authors independently extracted the following data from each of the eligible publications: presentation with DVT and/or PE, treatment characteristics, number of patients per group and incidence of recurrent fatal/non-fatal VTE per group during the period of comparison between placebo, ‘no treatment’ or ‘less intensive treatment’. Differences in opinion or interpretation were resolved by consensus.

### Statistical analysis

The incidences of recurrent VTE for the different treatment arms were used to calculate a separate odds ratio for each trial. These odds ratios were then combined across studies, giving weight to the number of events in each of the two treatment groups in each separate study, using the Mantel–Haenszel procedure [21], which assumes a fixed treatment effect. In addition, risk ratios were calculated using the metafor package in ‘R’. Likewise, 95% confidence intervals (CIs) for the effect estimate were calculated, and heterogeneity was statistically assessed. From the observed efficacy estimates and the lower limits of the CIs, thresholds were calculated for the retention of 50%, 66% and 75% of the treatment effect on both arithmetic and geometric scales.

## Results

A total of 5388 publications were identified in our literature search, which used the seminal publication of Barritt and Jordan as a starting point [7]. Review of the identified publications in accordance with the eligibility criteria resulted in the selection of a further 14 studies. One study was excluded because the ‘less intensive treatment’ regimen had been shown to be superior to placebo in another study [22,23]. Of the 14 studies included, nine evaluated anticoagulant therapy for a duration of up to 3 months [7-15], three for a duration of up to 12 months, [16,17,19] and two for longer durations [18,20].

Current standard of care was compared with placebo in five studies, with ‘no treatment’ in three studies and with ‘less intensive treatment’ in six studies. The design characteristics of these studies are summarised in Table 1. The results per study are summarised in Table 2.

**Table 1:** Summary of design characteristics for the 14 selected studies

**Study**	**Patient population**	**Acute therapy**	**Long-term therapy**	**Experimental treatment duration**	**Window for analysis of recurrent VTE**
Barritt [7]	PE	Intravenous UFH q6h vs placebo	VKA. PT 2.0–3.0 times control vs placebo	14 days	3 months
Hull [8]	DVT	Intravenous UFH	VKA started on day 10, PT 1.5–2.0 vs subcutaneous UFH bid	6 weeks for calf DVT and 12 weeks for proximal DVT	3 months
Holmgren [9]	DVT	UFH	VKA started on day 1, TT 5–14%	1 month vs 6 months	3 months
Lagerstedt [10]	Isolated calf DVT	Intravenous UFH	VKA started on day 1, INR 2.5–4.2 vs placebo	3 months	3 months
Hull [11]	Proximal DVT	Intravenous UFH vs subcutaneous UFH	VKA started on day 6 or 7, INR 2.0–3.0	3 months	3 months
Brandjes [12]	Proximal DVT	Intravenous UFH vs placebo	VKA started on day 1, INR 2.0–3.0	6 months	3 months
Raschke [13]	DVT or PE	Intravenous weight-based UFH vs ‘standard care’ UFH nomogram	VKA started after day 2	Not specified or monitored	3 months
Levine [14]	Proximal DVT	Intravenous UFH	VKA INR 2.0–3.0 vs placebo	4 weeks vs 12 weeks	3 months
Schulman [15]	DVT or PE	Intravenous UFH or subcutaneous LMWH	VKA started on day 1, INR 2.00–2.85	6 weeks vs 6 months	3 months
Agnelli [16]	Idiopathic proximal DVT	Intravenous UFH or subcutaneous LMWH	VKA INR 2.0–3.0 vs no treatment	Months 4–12 vs no treatment	Months 4–12
Agnelli [17]	PE	Intravenous UFH or subcutaneous LMWH	VKA INR 2.0–3.0 vs no treatment	Months 4–12 vs no treatment	Months 4–12
Kearon [18]	Idiopathic DVT or PE	Intravenous UFH or subcutaneous LMWH	VKA INR 2.0–3.0 vs placebo	Months 4–24 vs no treatment	Months 4–24
Pinede [19]	DVT or PE	Intravenous UFH or subcutaneous LMWH	VKA INR 2.0–3.0	Months 4–6 vs no treatment	Months 4–6
Schulman [20]	First recurrent DVT or PE	Intravenous UFH or subcutaneous LMWH	VKA INR 2.00–2.85 vs no treatment	Indefinitely vs none	Month 6 onwards

bid: twice daily; DVT: deep vein thrombosis; INR: international normalised ratio; PE: pulmonary embolism; PT: prothrombin time; q6h: every 6 hours; TT: thrombin time; UFH: unfractionated heparin; VKA: vitamin K antagonist; VTE: venous thromboembolism.

**Table 2:** Recurrent venous thromboembolic events during the comparison period

**Study**	**Current standard of care, n/N (%)**	**Placebo, ‘no treatment’ or ‘less intensive treatment’, n/N (%)**
Barritt [7]*	0/16 (0.0)	11/19 (57.9)
Hull [8]^†^	0/33 (0.0)	6/35 (17.1)
Holmgren [9]^‡^	3/66 (4.5)	6/69 (8.7)
Lagerstedt [10]^†^	0/23 (0.0)	7/28 (25.0)
Hull [11]	3/58 (5.2)	11/57 (19.3)
Brandjes [12]^§^	2/60 (3.3)	10/60 (16.7)
Raschke [13]	2/41 (4.9)	8/32 (25.0)
Levine [14]	1/109 (0.9)	9/105 (8.6)
Schulman [15]^¶^	4/454 (0.9)	26/443 (5.9)
Agnelli [16]**	4/134 (3.0)	11/133 (8.3)
Agnelli [17]^††^	1/165 (0.6)	6/161 (3.7)
Kearon [18]	1/79 (1.3)	17/83 (20.5)
Pinede [19]^‡‡^	1/361 (0.3)	6/375 (1.6)
Schulman [20]	3/116 (2.6)	23/111 (20.7)
Total number of recurrent events across studies	25/1715 (1.5)	157/1711 (9.2)

*This includes one event in the no treatment group at 8 weeks. ^†^Only symptomatic recurrent events were considered. ^‡^Based on figure one of the publication. When one treatment arm was shorter than 3 months, then only events that accumulated up to 3 months were counted. ^§^Events after 3 months were excluded. ^¶^Data provided by author. **Only events between 3 and 12 months were included. ^††^Only events between 3 and 12 months were included for patients with idiopathic PE and between 3 and 6 months for patients with provoked PE. ^‡‡^Only events between weeks 6 and 12 for patients with isolated calf DVT and between 3 and 6 months for patients with proximal DVT and/or PE.

DVT: deep vein thrombosis; PE: pulmonary embolism.

### Establishing the non-inferiority margin

Recurrent VTE occurred in 25 (1.5%) of 1715 patients who received current standard of care and in 157 (9.2%) of 1711 patients who received placebo, ‘no treatment’ or ‘less intensive treatment’ (Table 2). Therefore, the observed treatment effect for prevention of recurrent VTE was associated with an odds ratio of 0.18 (95% CI 0.14−0.25; test for heterogeneity, p=0.87) and a risk ratio of 0.19 (95% CI 0.12−0.28; test for heterogeneity, p=0.85). Conversely, in the absence of current standard treatment, the risk of recurrent VTE increased with an odds ratio of 5.56 (95% CI 4.00−7.14) or a risk ratio of 5.26 (95% CI 3.57−8.33) (Table 3). Hence, the lower limits of the 95% CIs of the effect needed to be maintained relevant for the calculation of the non-inferiority thresholds were 4.00 for the odds ratio and 3.57 for the risk ratio (Table 3). Thus, to preserve 50% of the established treatment effect, the threshold for non-inferiority equalled 2.50 if calculated using an odds ratio and 2.29 if calculated using a risk ratio on a linear scale. Likewise, to preserve 75% of the treatment effect, these thresholds should be 1.75 and 1.64, respectively (Table 3). In Table 3, the limits on a geometric scale are also presented.

**Table 3:** Calculation of non-inferiority margins based on 14 eligible studies

**Measure of association**	**Odds ratio (95% CI)**	**Risk ratio (95% CI)**
Point estimate of current standard of care vs placebo, ‘no treatment’ or ‘less intensive treatment’	0.18 (0.14−0.25)	0.19 (0.12−0.28)
Reversed point estimate of current standard of care vs placebo, ‘no treatment’ or ‘less intensive treatment’	5.56 (4.00−7.14)	5.26 (3.57−8.33)
**Non-inferiority margins**	**Odds ratio based on arithmetic/geometric scale**	**Risk ratio based on arithmetic/geometric scale**
Limit for no preserved effect	4.00/4.00	3.57/3.57
Threshold for preservation of 50% of effect	2.50/2.00	2.29/1.89
Threshold for preservation of 66% of effect	2.00/1.60	1.86/1.54
Threshold for preservation of 75% of effect	1.75/1.41	1.64/1.37
Threshold for preservation of 100% of effect*	1.00/1.00	1.00/1.00

*Superiority versus current standard of care.

CI: confidence interval.

The point estimates of the relative treatment effects between studies with a treatment duration of 3 months and studies with a treatment duration of longer than 3 months were consistent with odds ratios of 0.18 (95% CI 0.11−0.32) and of 0.18 (95% CI 0.12−0.28), respectively.

## Discussion

Our systematic review of the medical literature demonstrates that the current standard treatment for VTE is highly efficacious, with a pooled odds ratio of 0.18 and a pooled risk ratio of 0.19, corresponding to a relative risk reduction of over 80%. Based on this review, non-inferiority margins of 2.50, 2.00 and 1.75 could be calculated using odd ratios, corresponding to maintenance of documented efficacy of current standard treatment of 50%, 66% and 75%, respectively, on a linear scale. In general, if the non-inferiority margins were based on a risk ratio, the margins would be lower than that based on the odds ratio, which would lead to somewhat larger sample sizes.

The meta-analysis on which these margins are based included a large variety of studies undertaken over a long time period (i.e. between 1960 and 2013). Our search yielded more than 5000 potential references for review. However, attempts to narrow the search resulted in the loss of pertinent articles known to the authors. Even using these broad search criteria, the pertinent reference of Barritt and Jordan was not found. Therefore, the approach was supplemented by an extensive manual search to identify all relevant studies. All 14 included studies had a randomised design, reported clinical outcomes and documented the incidence of recurrent VTE. Although all studies included a group of patients that received the current standard of care, the comparator varied between placebo and ‘no treatment’ or some form of anticoagulant treatment that lacked evidence of efficacy. Despite the lack of evidence of efficacy, it cannot be excluded that these ‘less intensive’ anticoagulant regimens offered some protection against recurrent VTE, which would make our estimates of effect and accompanying non-inferiority margins relatively conservative. No statistical heterogeneity was identified when combining the results of the 14 identified studies. Based on the consistency of the observed effect in all studies and the high number of patients in the meta- analysis, the 95% CIs around the observed summary treatment effect are relatively narrow. With the observed large risk reduction, the calculated non-inferiority margin to demonstrate maintenance of 50% of the treatment effect, a commonly accepted approach, is 2.50 on a linear scale, which might be viewed as unacceptable because it involves clinically important events.

On a geometric scale, the comparable number for the non-inferiority margin would be 2.00. From our calculations, it is apparent that calculating a summary relative risk and then applying a geometric scale results in more stringent non-inferiority margins (Table 3). Part of this effect is caused by the wider CI around the summary effect estimate when using risk ratios rather than odds ratios, as a result of the different underlying statistical models. The other important choice is using an arithmetic (linear) or geometric scale. When reasoning from the event rate in a ‘no treatment’ situation, the logical choice would be to use a geometric scale (relative reduction of outcome rates upon relative reduction). However, when clinically evaluating the allowed excess of outcome events versus current standard treatment, the starting point is the observed incidence on current standard treatment and the logical choice would be to use a linear scale.

Recent published studies that evaluated direct oral anticoagulants in patients with symptomatic DVT and/or PE used 1.80, 2.00, 2.75 and 2.85 as non-inferiority margins to calculate their sample size. For the EINSTEIN DVT and EINSTEIN PE studies that evaluated the direct Factor Xa inhibitor rivaroxaban, it was calculated that a total of 88 events were needed for each study to demonstrate non-inferiority versus standard of care with a margin of 2.0 [24,25]. The observed upper limits of the 95% CIs around the relative treatment effect were 1.04 in the EINSTEIN DVT study, 1.68 in the EINSTEIN PE study, and 1.19 for all patients in the EINSTEIN DVT and EINSTEIN PE studies, corresponding to a retention of treatment effect of 99%, 77% and 94%, respectively, on a linear scale. Using a geometric scale, these values become 97%, 63% and 87%, respectively. In the RE-COVER and RE-MEDY studies that evaluated the direct thrombin inhibitor dabigatran versus current standard treatment in patients with DVT and/or PE, it was calculated that 46 and 40 events were needed for each of the studies to demonstrate non-inferiority versus standard of care with a margin of 2.75 and 2.85, respectively [26,27]. The observed upper limits of the 95% CIs around the relative treatment effect were 1.84 in the RE-COVER trial and 2.64 in RE-MEDY trial, corresponding to retention of treatment effect of 72% and 45%, respectively, on a linear scale. Using a geometric scale, these values become 56% and 30%, respectively. For the AMPLIFY study that evaluated the direct Factor Xa inhibitor apixaban in patients with DVT and/or PE, it was calculated that a total of 123 events were needed to demonstrate non-inferiority versus standard of care with a margin of 1.80 [28]. The observed upper limits of the 95% CIs around the relative treatment effect were 1.26 in patients with DVT, 1.61 in patients with PE, and 1.18 for all patients, corresponding to a retention of treatment effect of 91%, 79% and 94%, respectively, on a linear scale. Using a geometric scale, these values become 83%, 65% and 88%, respectively. In the HOKUSAI-VTE study, evaluating the direct Factor Xa inhibitor edoxaban in patients with symptomatic DVT and/or PE, a non-inferiority margin of 1.50 has been adopted (clinicaltrials.gov: NCT00986154) [29].

In summary, the available studies allow the calculation of non-inferiority margins using a statistical approach that are appropriate for the evaluation of direct oral anticoagulants in patients with DVT and/or PE. Studies on direct oral anticoagulants that were conducted or are ongoing have adopted various margins and differ in the amount of retention of treatment effect that they have demonstrated.

## Abbreviations

CI: Confidence interval; DVT: Deep vein thrombosis; PE: Pulmonary embolism; VKA: Vitamin K antagonist; VTE: Venous thromboembolism.

## Competing interests

MHP: Has acted as a consultant for Bayer HealthCare, Sanofi-aventis, Boehringer Ingelheim, GlaxoSmithKline, Daiichi Sankyo, Leo Pharma, Thrombogenics, Pfizer. AWAL is an employee of Bayer Pharma AG.

## Authors’ contributions

MHP and AWAL performed the analysis and interpreted the data, were involved in drafting and revising the manuscript, and provided final approval for publication of the manuscript.
